# Supplementary material for: A Cyclopentanone Compound Attenuates the Over-Accumulation of Extracellular Matrix and Fibrosis in Diabetic Nephropathy via Downregulating the TGF-β/p38MAPK Axis
Source: Biomedicines. 2022 Dec 16;10(12):3270. doi: 10.3390/biomedicines10123270 (PMC9775671; doi:10.3390/biomedicines10123270)
Supplement: Supplementary file 1 [file biomedicines-10-03270-s001.zip › biomedicines-2054600-supplementary.pdf]

Supplementary Materials

Table S1. Primers used for transcript quantification qPCR.

Species Gene	Accession Numbers	Forward Primer (5'-3')	Reverse Primer (5'-3')
MS E-cadherin	NM_009864.3	F: CAAGGACAGCCTTCTTTTCG	R: TGGACTTCAGCGTCACTTTG
MS TGF-β1	NM_011577.2	F: CTTCAATACGTCAGACATTCGGG	R:GTAACGCCAGGAATTGTTGCTA
MS Col IV	NM_007734.2	F: GGTGGGAAAATGTGATCCTGG	R: GCCTACGGATGGTTCTCCCT
MS a-SMA	NM_007392.3	F: CTGACAGAGGCACCACTGAA	R: CATCTCCAGAGTCCAGCACA
MS FN	NM_001276409.1	F: GCTCAGCAAATCGTGCAGC	R: CCATAGCAGGTACAAACCAGG
MS rps16	NM_013647.2	F: CGTGCTTGTGCTCGGAGCTA	R: GCTCCTTGCCCAGAAGCAAA
HS E-cadherin	NM_001317186.1	F: CTACCAGCCCAAAGTGTGTG	R: GTGTTATCGTGATTATCCGTGA
HS TGF-β1	NM_000660.6	F: CTAATGGTGGAAACCCACAACG	R: TATCGCCAGGAATTGTTGCTG
HS Col IV	NM_001303110.1	F: GGGATGCTGTTGAAAGGTGAA	R: GGTGGTCCGGTAAATCCTGG
HS FN	NM_001306130.1	F: CGGTGGCTGTCAGTCAAAG	R: AAACCTCGGCTTCCTCCATAA
HS a-SMA	NM_007392.3	F: TTCAATGTCCCAGCCATGTA	R:GAAGGAATAGCCACGCTCAG
HS rps16	NM_001020	F: TGGTCTCATCAAGGTGAACG	R: AAGTGAGTTTTGAGTCACGA
Rn E-cadherin	NM_031334.1	F: CATCACAGTCAAACGGCATC	R: TGGGAAACGTGAGCAGCTCT
Rn TGF-β1	NM_021578.2	F: CAACAATTCCTGGCGTTACCTTG	R: CGAAAGCCCTGTATTCCGTCTCC
Rn Col IV	NM_001135009.1	F: ATGTCCAAGGAAACGAGCGG	R: GTTGTCCCCAGAGATAGGTG
Rn FN	NM_019143	F: GCCTGAACCAGCCTACGGAT	R: ATGACCACTGCCAAAGCCCAAG
Rn a-SMA	NM_031004	F: CACCATCGGGAATGAACGCTTC	R: CTGTCAGCAATGCCTGGGTA
Rn rps16	NM_001169146.1	F: AAGTCTTCGGACGCAAGAAA	R: TGCCCAGAAGCAGAACAG

Table S2. The docking parameters of H8 with TGF-β1, MAPKs, and AKT.

Core Target	PDB ID	Absolute Energy	Conf Number	Relative Energy	LibDock Score
p38MAPK	1bl7	67.752	15	8.0566	104.628
ERK	6ge0	67.797	11	8.1016	71.1367
JNK	4whz	69.9402	12	10.2448	101.828
AKT	3cqu	77.2302	14	17.5348	101.055
TGF-β1	1py5	75.7904	13	16.0951	113.137

Notes: Based on the simulation analysis, the parameters, such as the absolute energy, the number of docking postures (Conf Number), the relative energy, and the comprehensive score of Libdock (LibDock score), were obtained. The lower absolute and relative energy between the inhibitor and the docking site indicates a stronger inhibitory effect of the inhibitor on the target protein, and the greater the number of Conf Number manifestes a greater possibility of an interaction of the inhibitor with the site. Finally, a higher LibDock score indicates a stronger inhibitory effect of the inhibitor. It is not difficult to see that the absolute energy and relative energy of H8 and p38MAPK are relatively low, and the comprehensive scores are relative high, indicating that H8 not only has affinity for TGF-β1, but also has better affinity for downstream signal p38MAPK.

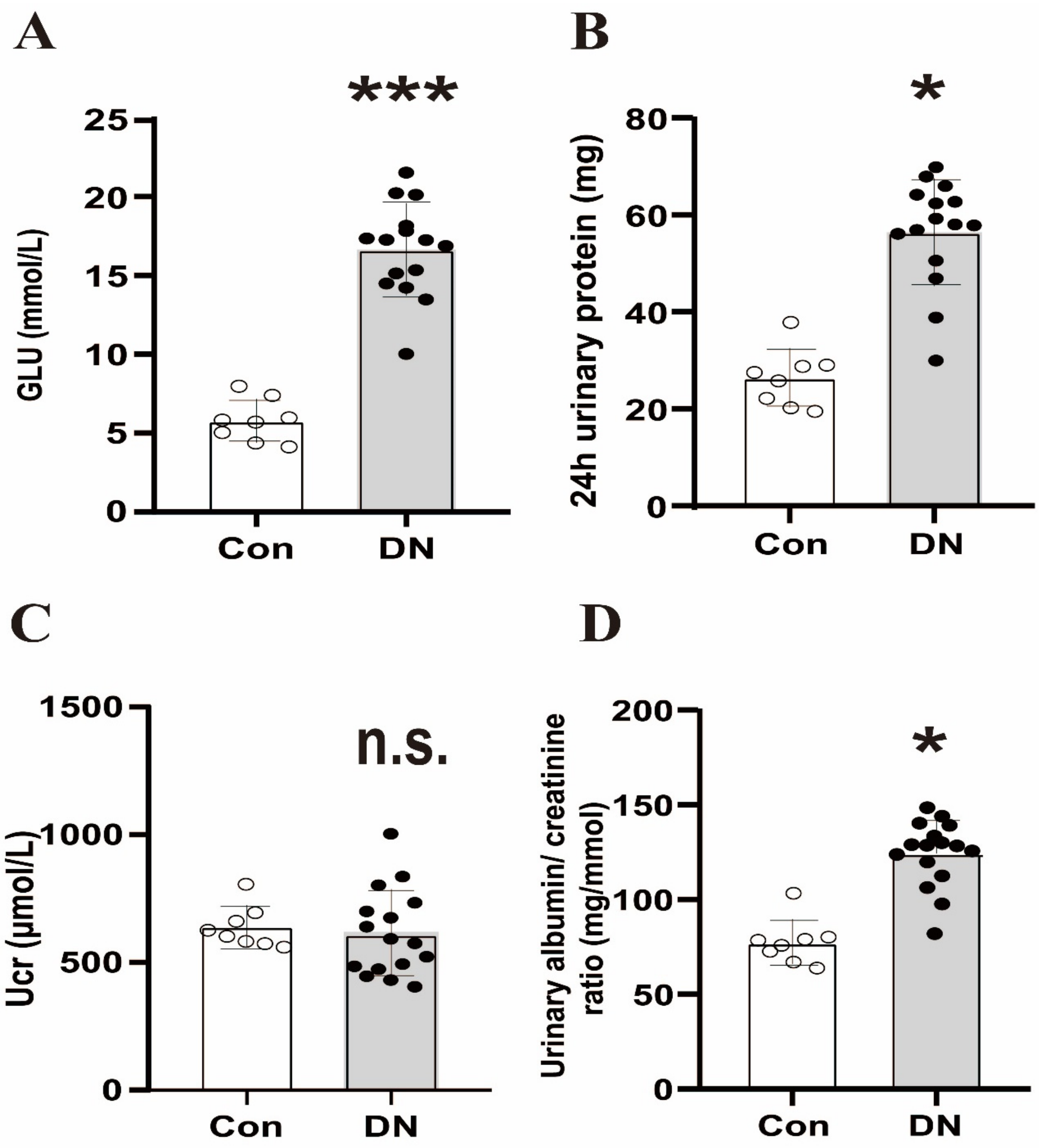

Figure S1. Identification of DN model rats. (**A**) GLU (mmol/L). (**B**) 24 h urinary protein (mg). (**C**) Ucr (μmol/L). (**D**) Urinary albumin/creatinine ratio (mg/mmol). Data are shown as mean ± S.D. (Con n = 8, diabetic rats n = 16). * $p < 0.05$, *** $p < 0.001$ compare to Con group. Con represents Control group and n.s. means no significant difference in statistics compared to Con group.

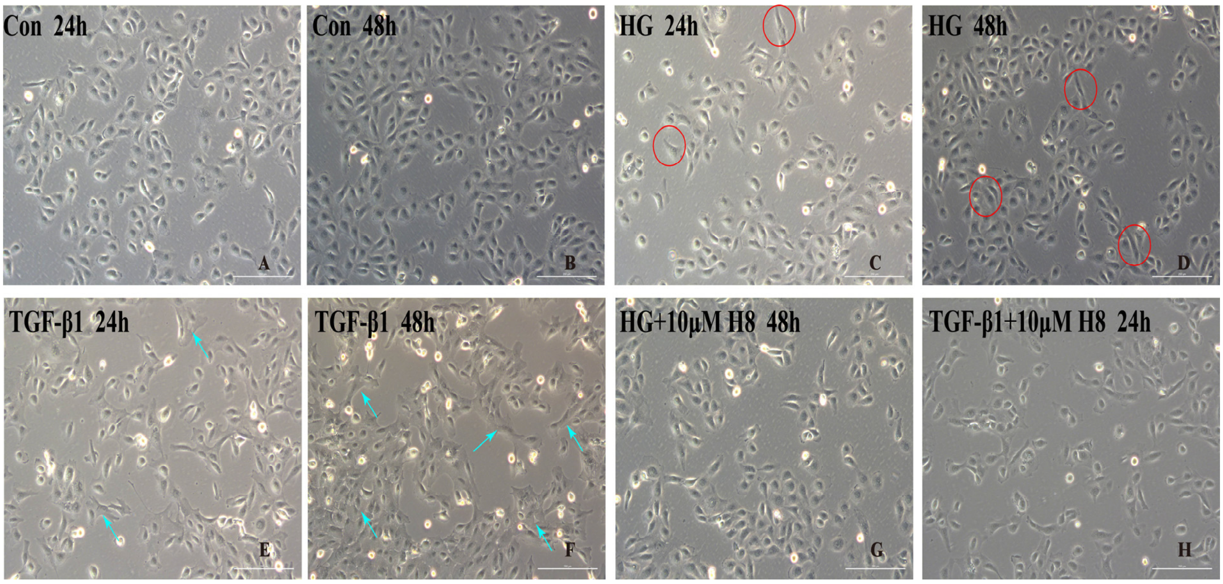

Figure S2. Cell morphology changes of damaged HK-2 cells. (**A**) Con group, 24 h. (**B**) Con group, 48 h. (**C**) HG group, 24 h. (**D**) HG group, 48 h. (**E**) TGF-β1 group, 24 h. (**F**) TGF-β1 group, 48 h. (**G**) HG+ 10 μM H8 group, 48 h. (**H**) TGF-β1+ 10 μM H8 group, 24 h. Red circle and blue arrows represent the damaged HK-2 cells; HG concentration: 35 mM; TGF-β1 concentration: 10 ng/mL; scale bars: 250 μm.
